# Dystonic Dysarthria in Wilson Disease: Efficacy of Zolpidem

**DOI:** 10.3389/fneur.2017.00559

**Published:** 2017-10-31

**Authors:** Aurélia Poujois, Michaela Pernon, Jean-Marc Trocello, France Woimant

**Affiliations:** ^1^National Reference Centre for Wilson Disease, Neurology Department, University Hospital Lariboisière, Assistance Publique-Hôpitaux de Paris, Paris, France

**Keywords:** Wilson disease, dysarthria, zolpidem, dystonia, voice therapy, imidazopyridine, benzodiazepines, copper

## Abstract

Wilson disease (WD) is a rare genetic disorder characterized by copper overload in the liver and the brain. Neurological presentations are mainly related to the accumulation of copper in the basal ganglia, the brainstem, and the cerebellum. Dysarthria is a frequent symptom, with dystonic, spastic, or parkinsonian components and is usually resistant to medical or voice rehabilitation therapies. Here, we report the case of a patient with WD diagnosed at the age of 12, who presented a severe and constant dysarthria from dystonic origin which was unresponsive to benzodiazepines and anticholinergic drugs. When she was 25-year-old, she tried zolpidem at bedtime for sleeping difficulties and reported a paradoxical effect of this drug on her voice. To confirm the effect of zolpidem on her dystonic dysarthria, we realized a full evaluation of her dysarthria at baseline without zolpidem and after 4 days of treatment by 10 mg twice a day. Lexical access was evaluated by the semantic fluency; dysarthria by the Intelligibility Score, the spontaneous speech and reading rates, the maximum phonation time on the sustained vowel [a] and by a perceptive evaluation. Two hours after the intake of zolpidem, improvement of all the parameters tested, with the exception of the maximum phonation time, was observed. Semantic fluency increased by 59%, the spontaneous speech rate by 88% and the reading rate by 76%. General dystonia remained unchanged and the tolerance of zolpidem was satisfactory. Since then, the patient takes zolpidem 5 mg five times a day, and 4 years later shows persistent improvement in oral communication and a good drug tolerance. In this single-case study, we showed that regular daytime intake of zolpidem could have a persisting effect on a complex dystonic dysarthria that was resistant to usual medical treatments.

## Introduction

Wilson disease (WD) is a rare genetic disorder due to mutation of the ATP7B gene. Clinical prevalence is estimated between 1.2 and 2/100,000 (1) but recent genetic studies reports a higher prevalence of this autosomal recessive inherited disease, around 1/7,000 (2, 3). An incomplete penetrance of the gene or the presence of modifier genes could account for the difference. More than 600 mutations have been reported so far and lead to a copper overload (4). The defective ATP7B protein predominantly expressed in the liver, alters the copper homeostasis as it does no longer incorporate copper in Ceruloplasmin neither release copper into the bile which result in a toxic copper accumulation with cell death. In the absence of diagnosis and early treatment, the hepatic non-Ceruloplasmin-bound copper spills into the bloodstream and is sequestered in other organs such as the brain. WD consequently evolves toward a systemic disease and its clinical features can vary from an asymptomatic state to hepatic and neuropsychiatric manifestations. Neurological symptoms are related to the accumulation of copper in specific brain regions of the brain such as the basal ganglia, the brainstem, and the cerebellum (5). Symptoms may include Parkinsonism, dysarthria, tremor, dystonia, and cerebellar abnormalities. Dystonia may be focal or generalized. When it concerns the face, oromandibular dystonia is often associated with lingual dystonia, jaw-opening dystonia and a facial involvement which realizes a very evocative face called “risus sardonicus” (6). Dysarthria with dystonic features is frequent, up to 80% in the literature, and may lead to mutism or pseudo-anarthria (7). With early treatment (copper chelators or zinc salts), spectacular regressions of pronounced neurological symptomatology may be observed but symptoms as dystonic dysarthria may persist and resist to anticholinergics drugs, tetrabenazine, baclofen, and benzodiazepines (8). In this single-case study, we present a patient with a severe dystonic type dysarthria who experienced a real and prolonged improvement of her voice after treatment by zolpidem.

## Patient Presentation

**Figure 1:**
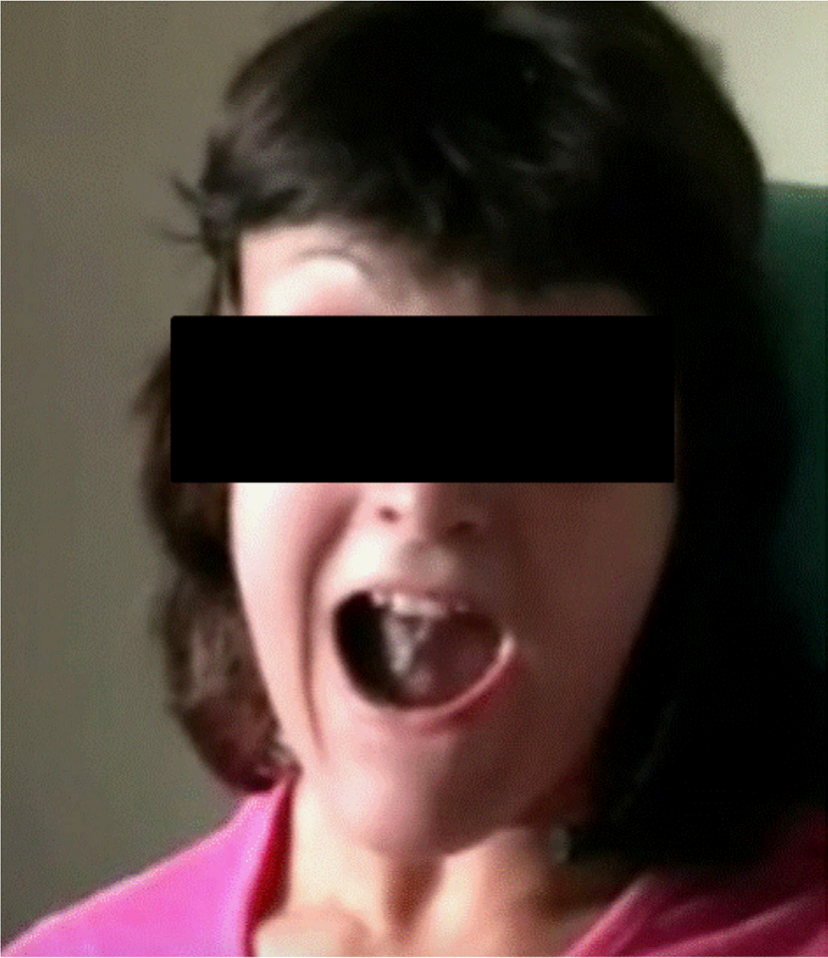
Oromandibular and lingual dystonia with jaw-opening dystonia and overflow phenomenon when the patient tries to speak.

A 25-year-old woman suffered from WD diagnosed at the age of 12 years. She had no family history of neurological or liver diseases. Her motor and intellectual development was normal until the age of 12 when she started to present behavioral troubles at school with signs of irritability, apathy alternating with aggressiveness. A few months later, a dysarthria appeared, rapidly followed by a generalized dystonia. Patient became rapidly mutic due to a major oro-facial dystonia and presented severe dysphagia requiring a jejunostomy. Brain MRI displayed bilateral T2 hypersignal in the putamen, the pallidum, the thalamus, and the mesencephalon. The presence of an abnormal copper balance with low cupremia, low ceruloplasminemia and excessive 24-h urinary copper excretion suggested the diagnosis of WD, especially since Kayser–Fleischer rings were present. Treatment by d-Penicillamine was initiated without delay pending the results of the molecular biology analysis of the ATP7B gene. WD diagnosis was confirmed 3 months later with the presence of two pathogenic mutations (Thr977Met in exon 13 and His1069Gln in exon 14) of the ATP7B gene. During the follow-up, her clinical symptoms progressively improved and the initial brain MRI lesions vanished while a basal ganglia T2 hyposignal appeared. d-Penicillamine was changed for another chelator, the triethylenetetramine following the appearance of a renal side effect (nephrotic syndrome). At the age of 25, her neurological condition was stabilized and her UWDRS (Unified WD Rating scale) scored 49/192 (9). She had a moderate generalized dystonia and could walk and eat without help. She had no more jejunostomy. Patient remained anarthric due to the persistence of an oromandibular and lingual dystonia that was triggered by the slightest movement or speech, preventing her for speaking (Figure 1). Botulinum toxin injections had improved cervical dystonia while benzodiazepines and anticholinergics were usefulness. Speech therapy was discontinued as it was ineffective. At this time, she tried zolpidem at bedtime for sleeping troubles and reported a paradoxical effect of this drug on her voice.

To document the effect of zolpidem, we realized a full assessment of her dysarthria: the first evaluation was made at baseline, 1 month after stopping zolpidem (video 1) and the second was done after 4 days of treatment by 10 mg twice a day (video 2). The video 2 was done 2 h after the intake of zolpidem. Lexical access was evaluated by the semantic fluency. Dysarthria was tested by the Intelligibility Score (IS, French adaptation of the Frenchay Dysarthria Assessment) (10), the spontaneous speech and reading rates, the maximum phonation time on the sustained vowel [a], and by a perceptive evaluation (PE) (11). Two hours after the intake of zolpidem, a dramatic improvement of all the tested parameters was observed, except for the maximum phonation time. Semantic fluency increased by 59%, the spontaneous speech rate by 88%, and the reading rate by 76% (Table 1). General dystonia remained unchanged as well as the UWDRS score, and the tolerance of zolpidem was satisfactory, without drowsiness, dizziness, or headache. Since then, the patient takes zolpidem 5 mg five times a day, and 4 years later shows persistent improvement in oral communication and a good drug tolerance. At that time, the patient refused to undergo a full evaluation without zolpidem as she could not speak a word without it, but she accepted to have a new evaluation 2 h after the intake of zolpidem. The assessment confirmed the sustained effect of zolpidem on her voice: maintenance of the semantic fluency score and the spontaneous speech rate and improvement of the IS, the maximum phonation time (due to a better pneumophonic coordination), the reading rate and the PE (Table 1). The patient noticed that the effect of zolpidem on her dysarthria remained stable over the past 4 years: it appears about 45 min after taking the medication and lasts 3 h.

**Table 1:** Speech evaluation: semantic fluency and dysarthria at initial evaluation and four years later.

	Initial evaluation		4 years later
	Without Zolpidem	2 h after zolpidem	2 h after zolpidem
**Lexical access**			
Semantic fluency	7	17	17
**Dysarthria**			
Intelligibility score (IS)			
Words	0	3	8
Sentences	0	4	2
Conversation	2	6	6
Total IS score	2	13	16
Categorization of Dysarthria	Severe	Moderate	Moderate
Spontaneous speech rate			
Words	5	39	24
Syllables	6	50	28
Syllables/second	0.2	1.7	1.5
Reading rate			
Words	6	154	134
Syllables	8	205	165
Syllables/second	0.24	1	1.4
Maximum phonation time	2.6	2.9	5
Perceptive evaluation (PE)			
Vocal quality	2	2	1
Phonetic realization	4	2	2
Prosodia	3	2	2
Breathing	3	2	1
Intelligibility	4	2	2
Natural characteristic	4	3	3
Total PE score	20	13	11

## Discussion and Conclusion

Dysarthria is a main problem in patients with neurological WD. This symptom is extremely frequent at the onset of the disease, being a “cardinal feature” of WD. Based on the analysis of 361 neurologic patients at diagnosis, dysarthria was present in 79.7% of patients and was the first symptom in 46% of them (8). Moreover, it usually takes a long time to improve and alters the patient’s possibilities of verbal communication. Physiopathology of dysarthria is related to the alteration of the basal ganglia circuitry, the brainstem, or the cerebellum by copper lesions, but may also be due to the dysfunction of the connectivity between multiple brain structures (12). Its clinical description is complex as WD dysarthria could have dystonic, parkinsonian, spastic or cerebellar patterns that could be mixed together or isolated (13). In most WD patients, and it was the case in our patient who had a generalized dystonia, a facial dystonia and a dystonic dysarthria, the phenotype of dysarthria is equivalent to the clinical symptoms of WD (tremor, dystonic, mixed) (14).

Specific treatment of dysarthria is disappointing. Speech therapy is the main treatment proposed to patients and is adapted to the specific type of speech anomalies. It requires long and tedious exercise programs which may slightly improve dysarthria but long-term effects are poor so this therapy is abandoned by patients after a few years (15). A few single-case publications reported the effect of different anti-dystonic medications on dystonic dysarthria. Anticholinergics drugs, tetrabenazine, baclofen, antiepileptic drugs, and benzodiazepines were tried but outcome was reported as insufficient (8, 16). Surgical treatment has been proposed once and its indication remains exceptional. In 2013, Sidiropoulos et al. reported the case of a 29-year-old bedridden WD patient with generalized dystonia and severe dysarthria in whom pharmacologic treatment of dystonia was ineffective (gabapentin, benzodiazepines, botulinum toxin). After a bilateral deep brain stimulation of the pallidum, his dystonic features improved and the patient started to vocalize (17).

Zolpidem was studied in dystonia due to its muscle relaxant properties but its effect in this indication is still a subject of controversy. The imidazopyridine zolpidem is a short-acting hypnotic chemically distinct from benzodiazepines. It increases the activity of GABA, an inhibitory neurotransmitter, by binding with a high affinity to GABAA receptor, a benzodiazepine subtype receptor BZ1 (18). GABAA receptors are found in the sensorimotor cortical regions, and also in numerous regions altered in WD like the globus pallidus, the pons, the ventral thalamic complex, and the cerebellum. Zolpidem could enhance inhibitory pathways in the basal ganglia motor loop, accounting for the clinical improvement in dystonia (19).

Few studies reported improvement in different movement disorders (20–24) and in various type of dystonia (25, 26). It appears to be more efficient in generalized or hand dystonia (27). Until now its effect on speech abilities has not been studied and our report is the first to give a detailed evaluation of its action on semantic fluency and dysarthria with testing of the intelligibility, the spontaneous speech and reading rates and the maximum phonation time associated with a PE. The major improvement reported concerned the semantic fluency and the dysarthria severity. Moreover, after a 4-year follow-up, we showed that this effect could be maintained and even continue to improve. Of course, one could argue that the good results obtained after 4 years could be due to the natural evolution of the disease under chelators, as the patient did not undergo the tests without zolpidem. However, the patient was in a stabilized condition since a few years and the other symptoms like cervical dystonia did not improve. What is more startling is that the patient cervical and axial dystonia, scored by the UWDRS, did not change after taking 5 mg of zolpidem. One explanation could be that the dosage of the drug is too low to be effective on the severe cervical dystonia. Furthermore, the UWDRS is not enough sensitive to catch a moderate change in axial dystonia. Since then, two other WD patients have taken zolpidem for a dystonic dysarthria with the same satisfactory result. One of the patient noticed also an improvement of his foot dystonia in the same time.

The tolerability of zolpidem was satisfactory, even after 4 years of continuous treatment. Its particular neuropharmacological activity probably explains that it does not have the side effects of benzodiazepines. In particular, it was pointed out that zolpidem is well tolerated in adults and the elderly, and that tolerance, abuse, addiction, rebounding insomnia, and other withdrawal effects do not develop in relation to zolpidem administration (28).

In conclusion, our report underlines a spectacular improvement with zolpidem in a patient with severe dysarthria due to facial and lingual dystonic posture. This treatment appears to be a useful option for treating dystonic dysarthria in a secondary dystonia as WD.

## Ethics Statement

No investigations or interventions were performed outside routine clinical care for this patient. As this is a case report, without experimental intervention into routine care, no formal research ethics approval was required. All the diagnostic, therapeutic, and video procedures were obtained with the written, fully informed consent of the patient. Verbal assent was also given by the patient himself. The authors confirm that the approval of an Institutional review board was not required for this work. The authors confirm that they have read the Journal’s position on issues involved in ethical publication and affirm that this work is consistent with those guidelines.

## Author Contributions

The authors declare to have given substantial contributions to the conception, acquisition, analysis, and interpretation of the manuscript. All authors have revised the manuscript critically and declared the final approval of the version to be published, and agreed to be accountable for all aspects of the work. AP: execution of the research project, review, critique, study supervision, and writing of the final manuscript. MP: execution of the research project and writing of the first draft. J-MT: conception, organization, and execution of the research project, writing of the first draft. FW: conception of the research project, review and critique of the manuscript.

## Conflict of Interest Statement

The authors declare that the research was conducted in the absence of any commercial or financial relationships that could be construed as a potential conflict of interest.

## Supplementary Material

Video S1 Evaluation of the dysarthria of the patient without zolpidem. Maximum phonation time of a sustained [a] was very short. Reading and spontaneous speech were limited to some words.

Video S2 Evaluation of the dysarthria of the patient 2 h after zolpidem. Maximum phonation time of a sustained [a] was longer. Reading and spontaneous speech rates improved as well as the intelligibility.
